# Pangenomics and Crop Genome Adaptation in a Changing Climate

**DOI:** 10.3390/plants11151949

**Published:** 2022-07-27

**Authors:** Jakob Petereit, Philipp E. Bayer, William J. W. Thomas, Cassandria G. Tay Fernandez, Junrey Amas, Yueqi Zhang, Jacqueline Batley, David Edwards

**Affiliations:** School of Biological Sciences, The University of Western Australia, Perth 6009, Australia; jakob.petereit@uwa.edu.au (J.P.); philipp.bayer@uwa.edu.au (P.E.B.); william.thomas@research.uwa.edu.au (W.J.W.T.); cassandria.tayfernandez@research.uwa.edu.au (C.G.T.F.); junrey.amas@research.uwa.edu.au (J.A.); yueqi.zhang@uwa.edu.au (Y.Z.); jacqueline.batley@uwa.edu.au (J.B.)

**Keywords:** climate-resilient crops, pangenomes, genomic diversity

## Abstract

During crop domestication and breeding, wild plant species have been shaped into modern high-yield crops and adapted to the main agro-ecological regions. However, climate change will impact crop productivity in these regions, and agriculture needs to adapt to support future food production. On a global scale, crop wild relatives grow in more diverse environments than crop species, and so may host genes that could support the adaptation of crops to new and variable environments. Through identification of individuals with increased climate resilience we may gain a greater understanding of the genomic basis for this resilience and transfer this to crops. Pangenome analysis can help to identify the genes underlying stress responses in individuals harbouring untapped genomic diversity in crop wild relatives. The information gained from the analysis of these pangenomes can then be applied towards breeding climate resilience into existing crops or to re-domesticating crops, combining environmental adaptation traits with crop productivity.

## 1. Introduction

The planet will warm by more than 1.5 °C by 2030 [1], which will result in a higher frequency and severity of unpredictable weather conditions [2]. These changes will lead to increased biotic and abiotic stress in crops and an overall reduction in crop yield.

Climate change will affect the weather patterns and CO2 concentrations of distinct regions in contrasting ways, such as losses and gains of yield [3,4]. While warmer climates can prolong growing windows and open up previously unsuitable growing areas in mid-to-high latitudes for maize and wheat [5], rice yields are modelled to drop in tropical and low-latitude regions [5,6]. Between 1981 and 2009, global warming reduced wheat production by 5.2% in India [7], while wheat yield in Europe dropped by 2.5% between 1989 and 2009 [8]. Conversely, the yield of fruiting vegetables increased in the Czech Republic from 1961 to 2014 [9].

To meet the increased world food demand of 56% by 2050 [10], it is necessary to develop more resilient varieties of staple, emerging, and orphan crops. Traditionally, the primary focus of breeding is improving yield and yield-influencing traits such as flowering time, alkaloid content and pod indehiscence [11]. Climate change has led to an increased interest in associated traits including drought, heat, and biotic stress tolerance [2,12,13].

Plant genomes are dynamic, which results in a reservoir of genetic diversity that gives plants the ability to adapt to changing environments [14,15,16,17]. However, crop genome diversity was reduced throughout domestication and breeding, where specific traits were selected such as fruit size, simultaneous seed ripening, seed compaction, seed retention, increased seed size and rapid germination [18].

The genes and genomic diversity necessary to develop future crop varieties will go beyond the knowledge gained from single reference genomes [19]. In contrast to single genome references, a pangenome can capture the genomic diversity of a group of individuals. It characterises genes as core, present in all accessions, or variable, absent in at least one accession. Pangenomes have been constructed for many crops, including soybean [20,21], tomato [22], cotton [23], rice [24], chickpea [25], sorghum [26], sunflower [27], maize (Hufford et al., 2021) and canola [28,29,30], and provide an important resource for the breeding of climate-resilient crops.

This review will discuss the opportunities and challenges presented by the impact of domestication and breeding on crop genomes and highlight how pangenomes can support the production of new crop varieties, adapted to current and future climate change.

## 2. Main

### 2.1. Pangenomes Highlight Individuals with Unused Genomic Diversity

The generation of climate-resilient crop varieties is partially dependent on existing traits in the genomes of available germplasm. It is likely that available traits in existing cultivars are not sufficient to overcome the challenges of the future, and breeders will be required to harness the genetic diversity found in crop wild relatives (CWR).

**Figure 1:**
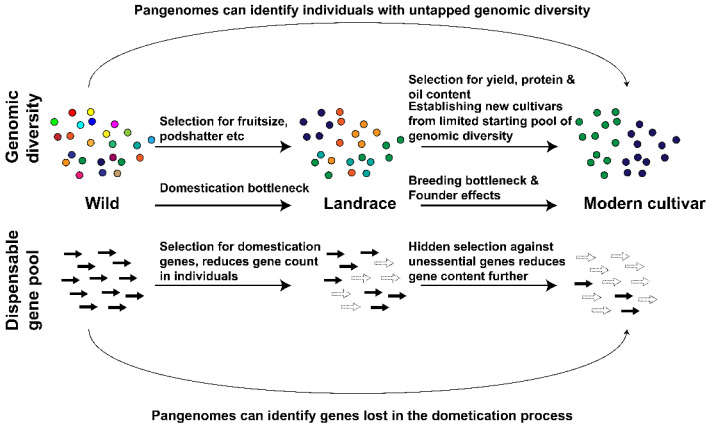
Effects of domestication and breeding bottlenecks on genomic diversity and dispensable genetic content.

The process of selecting plants showing agronomically favourable domestication traits, while at the same time narrowing their genetic background, is termed a domestication bottleneck [31] (Figure 1).

A domestication bottleneck has been described in maize, where a small founder population resulted in low genomic diversity after domestication [32]. However, domestication does not necessarily lead to a reduction in genomic diversity, as demonstrated by the domestication of Chinese soybean, which yielded approximately 45,000 landraces [33]. The significant soybean landrace variation could have served as broad genetic diversity to be used in the breeding of elite cultivars, yet the American soybean breeding programs only used 80 founding landraces for breeding programs [34]. More than 90% of the soybean genetic diversity found in the US is represented by the Williams and Lee cultivars [35]. This genetic bottleneck was extended in Brazilian soybean breeding programs which used a selection of American soybean lines as their founders, leading to a secondary founder effect [36]. Today, a single variety contributes up to 55.3% of the Brazilian soybean production [36], which introduces vulnerability to pathogens and a lack of resilience to changing climatic conditions.

Through domestication and breeding, wheat was selected for non-brittle rachis, naked grains [12], non-shattering grains [37] and for the Q allele mutation. The Q allele mutation affects a gene with pleiotropic effects on glume shape, glume tenacity and spike shape [38]. The Q mutation is present in all current naked hexaploid wheat varieties and is derived from a single ancestor [39]. Thus, hexaploid wheat has undergone many rounds of selection, introducing significant genetic bottlenecks and the associated loss of the diversity. Further founder effects and genetic bottlenecks can be observed in narrowleaf lupin [40], Nordic oat [41], barley [42], cassava [43], rice [44], maize [45]. Genetic bottlenecks are an unfortunate result of the domestication of most crops, and can reduce the reservoir of genetic variation that could be used to breed new varieties adapted to the changing climate.

Plants have been growing in diverse climatic conditions long before humanity started domesticating crops, moulding them towards current high yield elite varieties. Millions of years of evolution produced plants growing in cold, saline, dry, hot, or frequently changing environments [46,47,48] highlighting the potential of plants to adapt to changing climate. Diverse CWR stand in contrast to modern crops, that lost genes for resistance to biotic and abiotic stresses throughout domestication and breeding improvements (Figure 1). Lost genes are identified through pangenome studies that include wild accessions, and could potentially be reintroduced. For example, resistance to sclerotinia stem rot and soybean rust are characteristics of the perennial soybean *Glycine lattifolia*, and this trait is absent in the domesticated *Glycine max* [49,50]. However, these traits and their underlying genes can be reintroduced from wild relatives through breeding or genome editing.

Increased drought and heat stress are aspects of global warming that can reduce the productivity of crops. Global warming is predicted to reduce wheat yield by 6% and maize yield by 7.4% per degree Celsius increase in global mean temperature [51]. However, some crop varieties have been demonstrated to cope better with climate-related abiotic stress, which has been highlighted with the identification of 4 heat-resistant tomato varieties [52] and 23 potential heat-tolerant rice varieties [53], highlighting the potential to breed in adverse environments. 

### 2.2. Pangenomes Enable the Recovery of Genes Lost during Domestication and Breeding Improvements

Crop wild relatives can, due to their diverse gene pool, grow in environments that would cause significant stress for modern crop varieties. Pangenomes capture all genes within a population and can identify structural variations (SV), for example where a gene is present or absent in an individual, as well as gene copy number variation (CNV). By comparing the gene content of different domestication and breeding states in pangenome studies, it is possible to understand the impact of human selection on crop genomes.

The presence or absence of dispensable genes represents a measure of genomic diversity. Many dispensable genes demonstrate reduced frequency in populations following domestication [54,55], and sometimes genes are lost completely during domestication and breeding. The domestication process reduced the content of dispensable genes from 10.17% to 9.06% in soybean (Table 1) and from 20.98% to 18.6% in tomato (Table 1). Breeding improvements reduced the content of dispensable genes further to 8.69% in soybean, to 16.11% in tomato and reduced the dispensable gene content in cotton from 24.14% to 23.48% (Table 1).

Although dispensable genes are not essential for plant survival, they can contribute to plant fitness in specific environments. Dispensable genes are often enriched for functions involved in responses to biotic and abiotic stress, such as the defence response and the response to salt in soybean [54], the defence response, plant organ senescence and other defence-related processes in tomato [22], the response to environmental stress and defence response in bread wheat [56], defence to biotic stress and abiotic stress tolerance in rice [57] and disease resistance, defence response and water homeostasis in brassicas [28].

A breeding- and domestication-related reduction in the frequency of some genes and reduced dispensable gene content has been observed in some pangenome studies. The pangenome studies for soybean [54], cotton [23] and tomato [22] demonstrated that domestication and breeding can reduce the number of genes in modern elite crops compared to landraces or wild relatives. Gene content can be negatively correlated with yield, as shown for the NLR resistance gene family [58], and the selection for increased yield in modern elite crops may be leading to a reduced gene content. The loss of these genes may limit the ability of these crops to adapt to climate change scenarios, requiring the reintroduction of genes from wild relatives.

Presence–absence analysis in pangenomes can be used to identify genes that have been lost in modern crop varieties but are present in wild accessions or landraces. One example of stress tolerance is submergence tolerance in rice. Submergence tolerance was only present in a small number of landraces until a genetic study identified a major QTL on chromosome 9 that contributed significant submergence tolerance [59]. The QTL included three ethylene response transcription factors, including the SUB1A gene, which showed the allelic variation, SUB1A-1, that conferred submergence tolerance [60,61]. SUB1A-1 emerged in domesticated landraces through introgression from wild lines [62] and highlights the value of transferring genetic diversity from crop wild relatives into crops.

### 2.3. Accelerated Breeding and Targeted Genome Editing Can Alleviate the Impact of Reduced Genomic Diversity and Loss of Genes in Modern Cultivars

Genes identified in pangenomes that are absent from modern varieties help identify candidate genes that have potential for reintroduction into crops. While the identification of traits and underlying genes is accelerated through pangenome analysis and variation mapping, the conventional breeding of new crop varieties requires selection of complementary parental lines with desired traits, followed by crossing and the selection of progenies to develop a new cultivar [63]. The release of a new crop variety can take 10 years or more [64,65]. However, new approaches are being developed to accelerate the production of advanced varieties. The CRISPR/Cas system is a gene-editing technology that uses site-specific nucleases to bind and cleave nucleic acid sequences [66,67,68]. CRISPR/Cas9 has been adopted in crop species for yield improvement, improving biotic and abiotic stress resilience or adaptation to extreme environments [69,70]. The advance of this gene editing system and the diverse CRISPR variations, such as the Cas9 [71], Cas12a [72], fCas9 [73], RCas9 [74] and dCas systems [75,76], could help overcome some of the limitations caused by the diversity bottleneck and produce new gene variants and new traits that are not available in wild or domesticated germplasm [77].

CWR often have desirable traits that are not present in cultivated species, having been lost due to extensive domestication [78]. In addition to novel traits, CWR traits can be integrated back into modern elite crops using the CRISPR/Cas system. Among nearly 60,000 known species of CWRs, it is estimated that 10,000 can potentially contribute to breeding programs and therefore improve food security [79]. The extension of crop pangenomes from a species to wider phylogenetic groups can help identify additional genomic variation with potential for crop improvement [78]. For example, genetic variation differences between wild and cultivated soybean accessions were linked to agronomic traits such as oil content and biotic stress resistance [80]. Similarly, genetic diversity within 10 wild and 40 cultivated rice varieties were linked to domestication, flowering and disease resistance [81]. CWRs can be domesticated through CRISPR modification while retaining valuable wild-derived traits [82]. For example, Zsögön et al. (2018) genetically engineered 6 loci in the tomato wild relative *Solanum pimpinellifolium* known to be associated with yield. The newly domesticated variety had increased fruit size and number while retaining favourable, wild-derived high-nutritional content. In another study, Yu et al. [83] edited the loci controlling agronomically important traits using CRISPR/Cas9 in the allotetraploid rice wild relative *Oryza alta.* The altered variety had improved characteristics including reduced seed shattering, reduced awn length, reduced plant height, increased grain size, increased stem thickness and shortened heading date, while retaining the advantages of polyploidy such as genome buffering, vigour and environmental resilience. While CRISPR is a highly effective tool, the challenge of identifying target genes remains. This can be supported using pangenomes [84]. Pangenomes can be used as a reference for specific, multiplexed editing of SVs using CRISPR-Cas [84], allowing genes and traits of interest to be reintroduced without introducing deleterious traits [85].

Hybridisation and polyploidisation are major evolutionary forces that drive the evolution of novel phenotypes. For example, the allotetraploid species *Brassica napus* (oilseed rape; AACC) was formed from the natural interspecific hybridisation of *Brassica rapa* (AA) and *B. oleracea* (CC) ~7000 years ago [21]. However, just like most domesticated crops, intensive selection has resulted in a decrease in the genetic diversity of *B. napus*, making it vulnerable to biotic and abiotic stress. Efforts to recover lost genetic diversity have been carried out by resynthesising *B. napus* using the knowledge of the genetic relationship between the *Brassica* species (U 1935). Synthetic *B. napus* lines harbour more disease resistance genes [86], and are suggested to have wider ecological adaptation compared to their non-synthetic counterparts [87]. Furthermore, the development of allohexaploid *Brassica* species containing all the known *Brassica* subgenomes (A, B and C genomes: U 1935) have been reported from intermating diploid and tetraploid species [88]. These species are now referred to as “super-brassicas” which exhibit superior agronomic characteristics including climate-resilience traits [88]. Previously, breeders have been successful in breeding Triticale by crossing wheat and rye [89]. Synthetic polyploid cultivars have also been produced for other crops including watermelon, sugarbeet, grape, apple and banana [90].

The production of these new species is often challenging due to several factors. One major barrier for the successful establishment of new hybrid and polyploid species is meiotic instability [91]. In wheat, the major gene *Ph1* (pairing homoeologous) ensures a diploid-like chromosome pairing in offspring, stabilising their genomes [92]. However, meiotic stability is not well-characterised in other species; for example, in *Brassica* species where this trait is thought to be quantitatively controlled [93]. Thus, there is a requirement to describe the genetic factors controlling this trait for the production of new hybrid and polylploid crops. Pangenomes that comprehensively catalogue these genetic factors, along with target domestication genes, will be important for advancing this route to expand the gene pool for climate resilience in crops. Moreover, the production of these new species can largely benefit from pangenomes as they can inform selection of parental cultivars carrying the widest gene repertoire to maintain maximum genetic diversity in subsequent generations.

## 3. Conclusions and Outlook

Changing climate, the increasing global population and adverse political events are rapidly decreasing global food security. Climate change can both benefit and harm crop production. The application of the next generation of crop breeding offers the potential to both alleviate the risk of crop failure as well as harvest the benefits in regions where the climate becomes more favourable for crop growth. This review highlights the reduction in genomic diversity and gene content as side effects of domestication and breeding of modern crop varieties, and that this limits their potential for adaptation to changing environments. Harnessing the genomic diversity present in crop wild relatives can play a key role in unlocking the growth potential of modern crops under increasing surface temperatures and increased frequency of severe weather events.

## Figures and Tables

**Table 1:** Content of dispensable genes derived from selected pangenomes for soybean, cotton and tomato evaluated across different breeding states.

Crop	Breeding State	Dispensable Genes	Reference
Soybean	Wild (*G. soja*)	10.17%	[54]
	Landrace (*G. max*)	9.06%	
	Modern cultivar (*G. max*)	8.69%	
Cotton	Landrace (*G. hirsutum*)	24.14%	[55]
	Modern cultivar (*G. hirsutum*)	23.48%	
Tomato	Wild (*S. pimpinellifolium*)	20.98%	[22]
	Landrace (*S. lycopersicum* var. *cerasiforme*)	18.60%	
	Modern cultivar (*S. lycopersicum* var. *lycopersicum*-Heirloom)	16.11%	

## Data Availability

Not applicable.

## References

[1] Xu Y., Ramanathan V., Victor D.G. (2018). Global Warming Will Happen Faster than we Think. https://www.nature.com/articles/d41586-018-07586-5.

[2] Abberton M., Batley J., Bentley A., Bryant J., Cai H., Cockram J., Costa de Oliveira A., Cseke L.J., Dempewolf H., De Pace C. (2016). Global agricultural intensification during climate change: A role for genomics. Plant Biotechnol. J..

[3] Batley J., Edwards D. (2016). The application of genomics and bioinformatics to accelerate crop improvement in a changing climate. Curr. Opin. Plant Biol..

[4] Rosenzweig C., Iglesius A., Yang X.-B., Epstein P.R., Chivian E. (2001). Climate change and extreme weather events-Implications for food production, plant diseases, and pests. Glob. Change Hum. Health.

[5] Pugh T., Müller C., Elliott J., Deryng D., Folberth C., Olin S., Schmid E., Arneth A. (2016). Climate analogues suggest limited potential for intensification of production on current croplands under climate change. Nat. Commun..

[6] Parry M., Rosenzweig C., Livermore M. (2005). Climate change, global food supply and risk of hunger. Philos. Trans. R. Soc. B Biol. Sci..

[7] Gupta R., Somanathan E., Dey S. (2017). Global warming and local air pollution have reduced wheat yields in India. Clim. Change.

[8] Moore Frances C., Lobell David B. (2015). The fingerprint of climate trends on European crop yields. Proc. Natl. Acad. Sci. USA.

[9] Potopová V., Zahradníček P., Štěpánek P., Türkott L., Farda A., Soukup J. (2017). The impacts of key adverse weather events on the field-grown vegetable yield variability in the Czech Republic from 1961 to 2014. Int. J. Climatol..

[10] van Dijk M., Morley T., Rau M.L., Saghai Y. (2021). A meta-analysis of projected global food demand and population at risk of hunger for the period 2010–2050. Nature Food.

[11] Elzinga J.A., Atlan A., Biere A., Gigord L., Weis A.E., Bernasconi G. (2007). Time after time: Flowering phenology and biotic interactions. Trends Ecol. Evol..

[12] Doebley J.F., Gaut B.S., Smith B.D. (2006). The molecular genetics of crop domestication. Cell.

[13] Gepts P. (2004). Crop domestication as a long-term selection experiment. Plant Breeding Reviews.

[14] Adams K.L., Wendel J.F. (2005). Polyploidy and genome evolution in plants. Curr. Opin. Plant Biol..

[15] Kejnovsky E., Hawkins J., Feschotte C., Wendel J., Greilhuber J., Dolezel J., Leitch I. (2012). Plant Genome Diversity.

[16] Raza A., Razzaq A., Mehmood S.S., Zou X., Zhang X., Lv Y., Xu J. (2019). Impact of climate change on crops adaptation and strategies to tackle its outcome: A review. Plants.

[17] Wu X., Cai K., Zhang G., Zeng F. (2017). Metabolite profiling of barley grains subjected to water stress: To explain the genotypic difference in drought-induced impacts on malting quality. Front. Plant Sci..

[18] Zeder M.A., Emshwiller E., Smith B.D., Bradley D.G. (2006). Documenting domestication: The intersection of genetics and archaeology. Trends Genet..

[19] Bayer P.E., Golicz A.A., Scheben A., Batley J., Edwards D. (2020). Plant pan-genomes are the new reference. Nat. Plants.

[20] Bayer P.E., Valliyodan B., Hu H., Marsh J.I., Yuan Y., Vuong T.D., Patil G., Song Q., Batley J., Varshney R.K. (2021). Sequencing the USDA core soybean collection reveals gene loss during domestication and breeding. Plant Genome.

[21] Liu Y., Du H., Li P., Shen Y., Peng H., Liu S., Zhou G.-A., Zhang H., Liu Z., Shi M. (2020). Pan-Genome of Wild and Cultivated Soybeans. Cell.

[22] Gao L., Gonda I., Sun H., Ma Q., Bao K., Tieman D.M., Burzynski-Chang E.A., Fish T.L., Stromberg K.A., Sacks G.L. (2019). The tomato pan-genome uncovers new genes and a rare allele regulating fruit flavor. Nat. Genet..

[23] Li J., Yuan D., Wang P., Wang Q., Sun M., Liu Z., Si H., Xu Z., Ma Y., Zhang B. (2021). Cotton pan-genome retrieves the lost sequences and genes during domestication and selection. Genome Biol..

[24] Zhao Q., Feng Q., Lu H., Li Y., Wang A., Tian Q., Zhan Q., Lu Y., Zhang L., Huang T. (2018). Pan-genome analysis highlights the extent of genomic variation in cultivated and wild rice. Nat. Genet..

[25] Varshney R.K., Roorkiwal M., Sun S., Bajaj P., Chitikineni A., Thudi M., Singh N.P., Du X., Upadhyaya H.D., Khan A.W. (2021). A chickpea genetic variation map based on the sequencing of 3366 genomes. Nature.

[26] Ruperao P., Thirunavukkarasu N., Gandham P., Selvanayagam S., Govindaraj M., Nebie B., Manyasa E., Gupta R., Das R.R., Odeny D.A. (2021). Sorghum Pan-Genome Explores the Functional Utility for Genomic-Assisted Breeding to Accelerate the Genetic Gain. Front. Plant Sci..

[27] Hübner S., Bercovich N., Todesco M., Mandel J.R., Odenheimer J., Ziegler E., Lee J.S., Baute G.J., Owens G.L., Grassa C.J. (2019). Sunflower pan-genome analysis shows that hybridization altered gene content and disease resistance. Nat. Plants.

[28] Hufford M.B., Seetharam A.S., Woodhouse M.R., Chougule K.M., Ou S., Liu J., Ricci W.A., Guo T., Olson A., Qiu Y. (2021). De novo assembly, annotation, and comparative analysis of 26 diverse maize genomes. bioRxiv.

[29] Golicz A.A., Bayer P.E., Barker G.C., Edger P.P., Kim H., Martinez P.A., Chan C.K.K., Severn-Ellis A., McCombie W.R., Parkin I.A.P. (2016). The pangenome of an agronomically important crop plant *Brassica oleracea*. Nat. Commun..

[30] Hurgobin B., Golicz A.A., Bayer P.E., Chan C.-K.K., Tirnaz S., Dolatabadian A., Schiessl S.V., Samans B., Montenegro J.D., Parkin I.A.P. (2018). Homoeologous exchange is a major cause of gene presence/absence variation in the amphidiploid *Brassica napus*. Plant Biotechnol. J..

[31] Bayer P.E., Scheben A., Golicz A.A., Yuan Y., Faure S., Lee H., Chawla H.S., Anderson R., Bancroft I., Raman H. (2021). Modelling of gene loss propensity in the pangenomes of three *Brassica* species suggests different mechanisms between polyploids and diploids. Plant Biotechnol. J..

[32] Khush G.S. (2001). Green revolution: The way forward. Nat. Rev. Genet..

[33] Eyre-Walker A., Gaut R.L., Hilton H., Feldman D.L., Gaut B.S. (1998). Investigation of the bottleneck leading to the domestication of maize. Proc. Natl. Acad. Sci. USA.

[34] Hyten D.L., Song Q., Zhu Y., Choi I.-Y., Nelson R.L., Costa J.M., Specht J.E., Shoemaker R.C., Cregan P.B. (2006). Impacts of genetic bottlenecks on soybean genome diversity. Proc. Natl. Acad. Sci. USA.

[35] Carter T.E., Nelson R.L., Sneller C.H., Cui Z. (2004). Genetic diversity in soybean. Soybeans Improv. Prod. Uses.

[36] Valliyodan B., Cannon S.B., Bayer P.E., Shu S., Brown A.V., Ren L., Jenkins J., Chung C.Y.-L., Chan T.-F., Daum C.G. (2019). Construction and comparison of three reference-quality genome assemblies for soybean. Plant J..

[37] Wysmierski P.T., Vello N.A. (2013). The genetic base of Brazilian soybean cultivars: Evolution over time and breeding implications. Genet. Mol. Biol..

[38] Sood S., Kuraparthy V., Bai G., Gill B.S. (2009). The major threshability genes soft glume (sog) and tenacious glume (Tg), of diploid and polyploid wheat, trace their origin to independent mutations at non-orthologous loci. Theor. Appl. Genet..

[39] Simons K.J., Fellers J.P., Trick H.N., Zhang Z., Tai Y.-S., Gill B.S., Faris J.D. (2006). Molecular characterization of the major wheat domestication gene Q. Genetics.

[40] Charmet G. (2011). Wheat domestication: Lessons for the future. Comptes Rendus Biol..

[41] Berger J.D., Buirchell B.J., Luckett D.J., Nelson M.N. (2012). Domestication bottlenecks limit genetic diversity and constrain adaptation in narrow-leafed lupin *(Lupinus angustifolius* L.). Theor. Appl. Genet..

[42] Grau Nersting L., Bode Andersen S., von Bothmer R., Gullord M., Bagger Jørgensen R. (2006). Morphological and molecular diversity of Nordic oat through one hundred years of breeding. Euphytica.

[43] Parzies H., Spoor W., Ennos R. (2000). Genetic diversity of barley landrace accessions (*Hordeum vulgare* ssp. vulgare) conserved for different lengths of time in ex situ gene banks. Heredity.

[44] Peroni N., Hanazaki N. (2002). Current and lost diversity of cultivated varieties, especially cassava, under swidden cultivation systems in the Brazilian Atlantic Forest. Agric. Ecosyst. Environ..

[45] Prashanth S., Parani M., Mohanty B., Talame V., Tuberosa R., Parida A. (2002). Genetic diversity in cultivars and landraces of *Oryza sativa* subsp. indica as revealed by AFLP markers. Genome.

[46] Pressoir G., Berthaud J. (2004). Patterns of population structure in maize landraces from the Central Valleys of Oaxaca in Mexico. Heredity.

[47] Alberdi M., Bravo L.A., Gutiérrez A., Gidekel M., Corcuera L.J. (2002). Ecophysiology of Antarctic vascular plants. Physiol. Plant..

[48] Amtmann A., Bohnert H.J., Bressan R.A. (2005). Abiotic stress and plant genome evolution. Search for new models. Plant Physiol..

[49] Von Willert D., Eller B., Werger M., Brinckmann E. (1990). Desert succulents and their life strategies. Vegetatio.

[50] Hartman G., Gardner M., Hymowitz T., Naidoo G. (2000). Evaluation of perennial Glycine species for resistance to soybean fungal pathogens that cause Sclerotinia stem rot and sudden death syndrome. Crop Sci..

[51] Wen L., Yuan C., Herman T., Hartman G. (2017). Accessions of perennial Glycine species with resistance to multiple types of soybean cyst nematode (*Heterodera glycines*). Plant Dis..

[52] Zhao C., Liu B., Piao S., Wang X., Lobell D.B., Huang Y., Huang M., Yao Y., Bassu S., Ciais P. (2017). Temperature increase reduces global yields of major crops in four independent estimates. Proc. Natl. Acad. Sci. USA.

[53] Ayenan M.A.T., Danquah A., Hanson P., Asante I.K., Danquah E.Y. (2021). Identification of new sources of heat tolerance in cultivated and wild tomatoes. Euphytica.

[54] Tenorio F., Ye C., Redoña E., Sierra S., Laza M., Argayoso M. (2013). Screening rice genetic resources for heat tolerance. Sabrao J. Breed. Genet..

[55] Li G., Wang L., Yang J., He H., Jin H., Li X., Ren T., Ren Z., Li F., Han X. (2021). A high-quality genome assembly highlights rye genomic characteristics and agronomically important genes. Nat. Genet..

[56] Montenegro J.D., Golicz A.A., Bayer P.E., Hurgobin B., Lee H., Chan C.K.K., Visendi P., Lai K., Doležel J., Batley J. (2017). The pangenome of hexaploid bread wheat. Plant J..

[57] Yao W., Li G., Zhao H., Wang G., Lian X., Xie W. (2015). Exploring the rice dispensable genome using a metagenome-like assembly strategy. Genome Biol..

[58] Bayer P.E., Hu H., Petereit J., Derbyshire M.C., Varshney R.K., Valliyodan B., Nguyen H.T., Batley J., Edwards D. (2021). Yield is negatively correlated with nucleotide-binding leucine-rich repeat gene content in soybean. bioRxiv.

[59] Xu K., Mackill D.J. (1996). A major locus for submergence tolerance mapped on rice chromosome 9. Mol. Breed..

[60] Bailey-Serres J., Fukao T., Ronald P., Ismail A., Heuer S., Mackill D. (2010). Submergence tolerant rice: SUB1’s journey from landrace to modern cultivar. Rice.

[61] Xu K., Xu X., Fukao T., Canlas P., Maghirang-Rodriguez R., Heuer S., Ismail A.M., Bailey-Serres J., Ronald P.C., Mackill D.J. (2006). Sub1A is an ethylene-response-factor-like gene that confers submergence tolerance to rice. Nature.

[62] Pucciariello C., Perata P. (2013). Quiescence in rice submergence tolerance: An evolutionary hypothesis. Trends Plant Sci..

[63] Shimelis H., Laing M. (2012). Timelines in conventional crop improvement: Pre-breeding and breeding procedures. Aust. J. Crop Sci..

[64] Ahmar S., Gill R.A., Jung K.-H., Faheem A., Qasim M.U., Mubeen M., Zhou W. (2020). Conventional and molecular techniques from simple breeding to speed breeding in crop plants: Recent advances and future outlook. Int. J. Mol. Sci..

[65] Gerald N., Frei U.K., Lübberstedt T. (2013). Accelerating plant breeding. Trends Plant Sci..

[66] Osakabe Y., Osakabe K. (2015). Genome editing with engineered nucleases in plants. Plant Cell Physiol..

[67] Scheben A., Wolter F., Batley J., Puchta H., Edwards D. (2017). Towards CRISPR/Cas crops—Bringing together genomics and genome editing. New Phytol..

[68] Wang H., La Russa M., Qi L.S. (2016). CRISPR/Cas9 in Genome Editing and Beyond. Annu. Rev. Biochem..

[69] Marsh J.I., Hu H., Gill M., Batley J., Edwards D. (2021). Crop breeding for a changing climate: Integrating phenomics and genomics with bioinformatics. Theor. Appl. Genet..

[70] Warschefsky E., Penmetsa R.V., Cook D.R., von Wettberg E.J. (2014). Back to the wilds: Tapping evolutionary adaptations for resilient crops through systematic hybridization with crop wild relatives. Am. J. Bot..

[71] Ran F.A., Hsu P.D., Wright J., Agarwala V., Scott D.A., Zhang F. (2013). Genome engineering using the CRISPR-Cas9 system. Nat. Protoc..

[72] Bandyopadhyay A., Kancharla N., Javalkote V.S., Dasgupta S., Brutnell T.P. (2020). CRISPR-Cas12a (Cpf1): A Versatile Tool in the Plant Genome Editing Tool Box for Agricultural Advancement. Front. Plant Sci..

[73] Guilinger J.P., Thompson D.B., Liu D.R. (2014). Fusion of catalytically inactive Cas9 to FokI nuclease improves the specificity of genome modification. Nat. Biotechnol..

[74] Terns M.P. (2018). CRISPR-Based Technologies: Impact of RNA-Targeting Systems. Mol. Cell.

[75] Kazi T.A., Biswas S.R. (2021). CRISPR/dCas system as the modulator of gene expression. Prog. Mol. Biol. Transl. Sci..

[76] Zaidi S.S.-e.-A., Mahas A., Vanderschuren H., Mahfouz M.M. (2020). Engineering crops of the future: CRISPR approaches to develop climate-resilient and disease-resistant plants. Genome Biol..

[77] Scheben A., Edwards D. (2017). Genome editors take on crops. Science.

[78] Khan A.W., Garg V., Roorkiwal M., Golicz A.A., Edwards D., Varshney R.K. (2020). Super-Pangenome by Integrating the Wild Side of a Species for Accelerated Crop Improvement. Trends Plant Sci..

[79] Maxted N., Kell S. (2009). Establishment of a Global Network for the In Situ Conservation of Crop Wild Relatives: Status and Needs.

[80] Zhou Z., Jiang Y., Wang Z., Gou Z., Lyu J., Li W., Yu Y., Shu L., Zhao Y., Ma Y. (2015). Resequencing 302 wild and cultivated accessions identifies genes related to domestication and improvement in soybean. Nat. Biotechnol..

[81] Xu X., Liu X., Ge S., Jensen J.D., Hu F., Li X., Dong Y., Gutenkunst R.N., Fang L., Huang L. (2012). Resequencing 50 accessions of cultivated and wild rice yields markers for identifying agronomically important genes. Nat. Biotechnol..

[82] Gasparini K., Moreira J.d.R., Peres L.E.P., Zsögön A. (2021). *De novo* domestication of wild species to create crops with increased resilience and nutritional value. Curr. Opin. Plant Biol..

[83] Yu H., Lin T., Meng X., Du H., Zhang J., Liu G., Chen M., Jing Y., Kou L., Li X. (2021). A route to de novo domestication of wild allotetraploid rice. Cell.

[84] Tay Fernandez C.G., Nestor B.J., Danilevicz M.F., Marsh J.I., Petereit J., Bayer P.E., Batley J., Edwards D. (2022). Expanding Gene-Editing Potential in Crop Improvement with Pangenomes. Int. J. Mol. Sci..

[85] Bosse M., Megens H.J., Derks M.F.L., de Cara Á.M.R., Groenen M.A.M. (2019). Deleterious alleles in the context of domestication, inbreeding, and selection. Evol. Appl..

[86] Dolatabadian A., Bayer P.E., Tirnaz S., Hurgobin B., Edwards D., Batley J. (2020). Characterization of disease resistance genes in the *Brassica napus* pangenome reveals significant structural variation. Plant Biotechnol. J..

[87] Karim M.M., Siddika A., Tonu N.N., Hossain D.M., Meah M.B., Kawanabe T., Fujimoto R., Okazaki K. (2014). Production of high yield short duration *Brassica napus* by interspecific hybridization between *B. oleracea* and *B. rapa*. Breed Sci..

[88] Zhang K., Mason A.S., Farooq M.A., Islam F., Quezada-Martinez D., Hu D., Yang S., Zou J., Zhou W. (2021). Challenges and prospects for a potential allohexaploid *Brassica* crop. Theor. Appl. Genet..

[89] Stace C.A. (1987). Triticale: A Case of Nomenclatural mistreatment. TAXON.

[90] Ruiz M., Oustric J., Santini J., Morillon R. (2020). Synthetic Polyploidy in Grafted Crops. Front. Plant Sci..

[91] Mason A.S., Zhang J., Tollenaere R., Vasquez Teuber P., Dalton-Morgan J., Hu L., Yan G., Edwards D., Redden R., Batley J. (2015). High-throughput genotyping for species identification and diversity assessment in germplasm collections. Mol. Ecol. Resour..

[92] Rawale K.S., Khan M.A., Gill K.S. (2019). The novel function of the Ph1 gene to differentiate homologs from homoeologs evolved in Triticum turgidum ssp. dicoccoides via a dramatic meiosis-specific increase in the expression of the 5B copy of the C-Ph1 gene. Chromosoma.

[93] Higgins E.E., Howell E.C., Armstrong S.J., Parkin I.A.P. (2021). A major quantitative trait locus on chromosome A9, BnaPh1, controls homoeologous recombination in *Brassica napus*. New Phytol..

